# *RSPO1*-mutated keratinocytes from palmoplantar keratoderma display impaired differentiation, alteration of cell–cell adhesion, EMT-like phenotype and invasiveness properties: implications for squamous cell carcinoma susceptibility in patients with 46XX disorder of sexual development

**DOI:** 10.1186/s13023-022-02434-2

**Published:** 2022-07-19

**Authors:** Elena Dellambra, Sonia Cordisco, Francesca Delle Monache, Sergio Bondanza, Massimo Teson, Ezio Maria Nicodemi, Biagio Didona, Angelo Giuseppe Condorelli, Giovanna Camerino, Daniele Castiglia, Liliana Guerra

**Affiliations:** 1 Laboratory of Molecular and Cell Biology, Istituto Dermopatico Dell’Immacolata, IDI-IRCCS, Via dei Monti di Creta 104, 00167 Rome, Italy; 2 Advent SRL, Via Pontina KM 30.600, Pomezia, Italy; 3 Department of Life, Health and Environmental Sciences, University of L’Aquila, L’Aquila, Italy; 4 Center for Regenerative Medicine Stefano Ferrari, Holostem Terapie Avanzate S.R.L., 41125 Modena, Italy; 5 Plastic Surgery Division, Istituto Dermopatico Dell’Immacolata, IDI-IRCCS, Rome, Italy; 6 Rare Skin Disease Center, Istituto Dermopatico Dell’Immacolata, IDI-IRCCS, Rome, Italy; 7 Genodermatosis Unit, Genetics and Rare Diseases Research Division, Bambino Gesù Children’s Hospital, IRCCS, 00165 Rome, Italy; 8 Dipartimento di Patologia Umana ed Ereditaria, Sezione di Biologia Generale e Genetica Medica, Università Di Pavia, Via Forlanini 14, 27100 Pavia, Italy

**Keywords:** R-spondin, Palmoplantar keratoderma, Squamous cell carcinoma, Sex reversal, Primary keratinocytes

## Abstract

**Background:**

Secreted R-spondin (RSPO) proteins play a key role in reproductive organ development, epithelial stem cell renewal and cancer induction by reinforcing canonical Wnt signaling. We have previously reported that palmoplantar keratoderma (PPK), predisposition to cutaneous squamous cell carcinoma (SCC) development and sex reversal segregate as autosomal recessive trait in patients carrying *RSPO1*-mutations.

Although our previous findings suggested that RSPO1 secreted from fibroblasts regulates keratinocyte growth or differentiation, the role of this protein in the epidermis remains largely unexplored. Our study was aimed at expanding the phenotypic, molecular and functional characterization of *RSPO1*-mutated skin and keratinocytes.

**Results:**

Cultured primary keratinocytes from PPK skin of a *RSPO1*-mutated XX-sex reversed patient displayed highly impaired differentiation and epithelial-mesenchymal transition (EMT)-like phenotype. Interestingly, *RSPO1*-mutated PPK skin expressed markers of increased proliferation, dedifferentiation and altered cell–cell adhesion. Furthermore, all these signs were more evident in SCC specimens of the patient. Cultured PPK patient’s keratinocytes exhibited increased expression of cell‒matrix adhesion proteins and extracellular matrix remodeling enzymes. Moreover, they showed invasiveness properties in an organotypic skin model in presence of PPK fibroblasts, which behave like cancer-associated fibroblasts. However, the co-culture with normal fibroblasts or treatment with the recombinant RSPO1 protein did not revert or reduce the EMT-like phenotype and invasion capability of PPK keratinocytes. Notably, *RSPO1*-mutated PPK fibroblasts induced a hyperproliferative and dedifferentiated phenotype of age-matched normal control plantar keratinocytes. Wnt signaling has a key role in both PPK promotion and SCC development. Accordingly, Wnt mediators were differentially expressed in both PPK keratinocytes and skin specimens of *RSPO1*-mutated patient compared to control.

**Conclusions:**

Altogether our data indicate that the absence of RSPO1 in patients with 46XX disorder of sexual development affects the skin microenvironment and epidermal integrity, thus contributing to the risk of SCC tumorigenesis in palmoplantar regions exposed to major frictional stresses.

**Supplementary Information:**

The online version contains supplementary material available at 10.1186/s13023-022-02434-2.

## Background

Rspondins (RSPO) constitute a family of four secretory proteins (RSPO 1–4). They display a key role in embryonic development, epithelial stem cell renewal and cancer induction [1–6]. Germline *RSPO1* and *RSPO4* mutations result in developmental disorders involving reproductive organs and nail, respectively [7, 8].

Up to date, 9 individuals with constitutive *RSPO1* biallelic mutations have been reported. These individuals show 46XX disorders of sexual development and palmoplantar keratoderma (PPK) [3, 8–10]. All identified mutations are predicted to cause loss of protein function by affecting the highly conserved N-terminal furin-like cysteine-rich domains, which have a key role in WNTβ-catenin signaling activation [3, 8–15]. Notably, *RSPO1* loss-of-function mutations were also associated with squamous cell carcinoma (SCC) development in palmoplantar sites in 5 of the 9 patients so far described [3, 11–15].

PPK is a clinical feature of a heterogeneous group of acquired and hereditary disorders. It consists of persistent thickening of the epidermis over the palms and soles and is characterized by keratinocyte hyperproliferation and excessive production of horny layer (hyperkeratosis) that develop in response to an inherent fragility of the tissue or a dysregulation of cell proliferation. Indeed, proteins affected in hereditary PPK fulfill various functions, including mechanical stabilization (e.g. defects of keratins or desmosomal proteins), cell signaling and cell-to-cell communication (e.g. alterations of signaling, transport, receptors). Notably, some inherited forms of PPK are prone to SCC development [16, 17].

RSPO proteins positively regulate Wnt signaling in triggering self-renewal, proliferation, differentiation and morphogenesis. Furthermore, defects in Wnt pathway components have been associated with SCCs [1, 18]. RSPO proteins bind with high affinity Lgr4 and Lgr5 receptors, which are up-regulated in various cancers [19]. The binding potentiates canonical Wnt signaling by enhancing Wnt-induced LRP6 phosphorylation [4, 19]. RSPO-mediated stimulation of Wnt pathway results in the activation of β-catenin, a component of cell–cell adhesion junctions. When released from the complex with E-cadherin, β-catenin moves into the nucleus and stimulates the expression of target genes, mainly involved in driving the epithelial-to-mesenchymal transition (EMT) process that is directly associated with invasion and metastasis [20, 21].

RSPO1 isoform stimulates the proliferation of intestinal epithelial and corneal endothelial cells, and regulates hair follicle and mammary stem cell self-renewal [5, 20–24]. We have previously reported that RSPO1 is expressed in dermal fibroblasts but not in keratinocytes of normal skin [3]. Furthermore, cultured primary keratinocytes from PPK area of a *RSPO1*-mutated patient displayed an abnormal phenotype and were not able to differentiate in an organotypic skin model [3]. Very recently, we have demonstrated that fibroblasts obtained from PPK area of two patients with germline *RSPO1* mutations display a cancer-associated phenotype [25]. This finding indicates that RSPO1 released by dermal fibroblasts takes part in dermal-epidermal signaling, and its deficiency may account for abnormal keratinocyte behavior affecting epidermal homeostasis.

To increase knowledge of the pathophysiology of RSPO1 related disorders, in the present study we have deeply characterized the phenotypic, molecular and functional features of cutaneous keratinocytes from a *RSPO1*-mutated XX-sex reversed patient, who suffers from recurrent and malignant cutaneous SCCs. Our findings highlight a role for *RSPO1* mutations in cutaneous SCC susceptibility.

## Results

### Primary keratinocytes from affected plantar hyperkeratotic skin of *RSPO1*-mutated patient display impaired differentiation

Cultured keratinocytes reconstitute a stratified squamous epithelium which maintains all the characteristics as well as specific differentiation features of the original donor site [26].

**Fig. 1:**
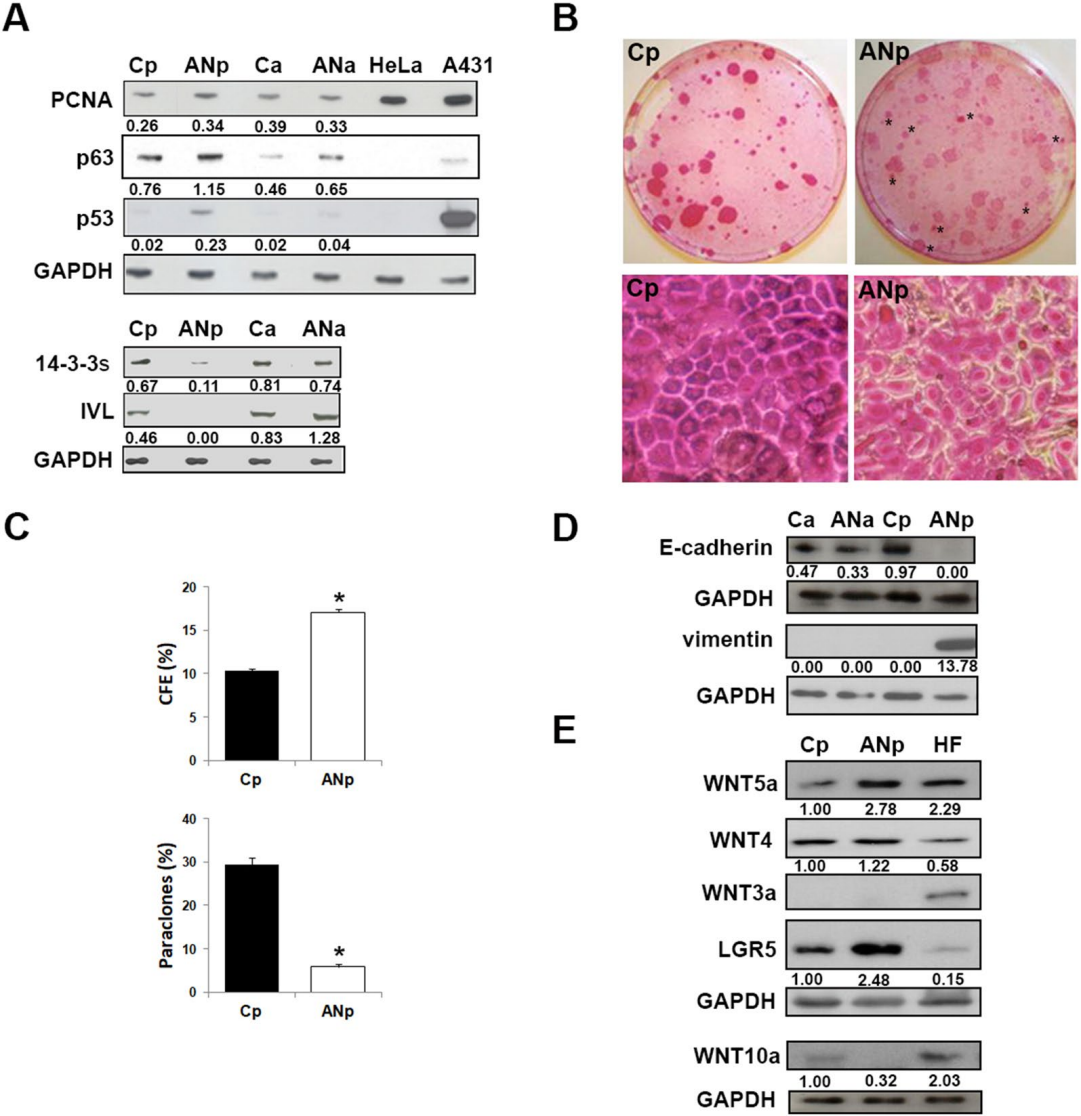
Keratinocytes from affected plantar hyperkeratosic skin of *RSPO1*-mutated patient displays differentiation impairment and EMT-like phenotype. **A** Immunoblots of PCNA, p63, p53, 14–3-3 sigma, involucrin (IVL) in keratinocytes from affected plantar (ANp) and unaffected abdominal (ANa) skin of *RSPO1*-mutated XX-sex reversed patient AN, and from plantar (Cp) and abdominal (Ca) skin of aged-matched unrelated donor. Densitometric values were normalized to GAPDH levels. HeLa and A431 cell lines were positive controls for PCNA and p53, respectively. **B** Representative images of colony forming efficiency (CFE) of Cp and ANp cultures. **C** CFE values and percentage of aborted colonies (paraclones) in Cp and ANp cultures (n = 3). Data are shown as mean ± SD. *P < 0.05. **D** Immunoblots of E-cadherin and vimentin in ANp, ANa, Cp and Ca keratinocyte cultures. Densitometric values were normalized to GAPDH levels. **E** Immunoblots of Wnt-3a, Wnt-4, Wnt5a, Wnt-10a and Lgr5 in ANp and Cp keratinocyte cultures. Densitometric values were normalized to GAPDH levels and expressed as fold change. Human fibroblasts (HF) were positive controls for Wnt-3a.

Higher expression of proliferation markers (p63, PCNA) was observed in primary keratinocytes cultured from PPK area of the *RSPO1*-mutated patient (ANp) compared to patient abdomen and control plantar (Cp) keratinocytes (Fig. 1A). ANp keratinocytes also showed a higher expression of p63 compared to cells from patient abdomen (ANa). ANp keratinocytes display a higher expression of p53, a protein playing a crucial role in tumor suppression, cell cycle, DNA repair and other pro-survival pathways. Besides promoting proliferation of basal keratinocytes, p63 also regulates keratinocyte differentiation and counteracts cell senescence [27]. Notably, strong down-regulation of some differentiation markers, such as involucrin and 14–3-3 sigma, was observed in ANp keratinocytes as compared to cells from Cp, ANa and control abdomen (Ca) areas.

We previously noted that *RSPO1*-mutated plantar keratinocytes were unable to correctly differentiate in an organotypic skin model [3]. Results here obtained indicate that the major impairment of the differentiation process is an inherent and partially site-specific feature of *RSPO1*-mutated patient keratinocytes.

### Primary keratinocytes from affected plantar hyperkeratotic skin of *RSPO1*-mutated patient display EMT-like phenotype

Cp were able to form colonies composed by small cells tightly adherent to each other. On the contrary, ANp keratinocytes were characterized by altered epithelial cell morphology and larger intercellular spaces as compared to control plantar cells [3]. This cell–cell dissociation suggests the presence of defects in intercellular junctions. Indeed, we previously detected reduced levels of β-catenin in cultured ANp keratinocytes respect to Cp [3]. In addition, ANp colonies displayed an EMT-like phenotype, similar to that of SCC cell lines (SCC-13) [28] or RAS-V12-transduced cultures [29]. No morphological differences were detected between ANa and Ca keratinocyte cultures (data not shown).

Colony forming efficiency (CFE) assay measures the clonogenic ability of keratinocytes. The percentage of paraclones, which contain terminally differentiated keratinocytes programmed for limited growth, is a parameter of senescence [30]. ANp keratinocytes had a significant increase of colony forming efficiency (CFE) and reduction of paraclone percentage compared to Cp (Fig. 1B, C). The assay also confirmed that Cp keratinocytes form stratified colonies (Fig. 1B, Cp). On the contrary, ANp keratinocytes formed only few stratified colonies (Fig. 1B, ANp, asterisks), with most keratinocytes growing in monolayer.

EMT is characterized by several phenotypic changes, including destabilization of cell–cell contacts, loss of cell polarity, shape modifications, cellular dedifferentiation, switch from keratin to vimentin intermediate filament expression. A hallmark of EMT is the downregulation of E-cadherin [31]. Highly reduced levels of the epithelial marker E-cadherin and increased levels of the mesenchymal marker vimentin were detected in patient plantar keratinocytes (ANp), in keeping with their EMT-like phenotype (Fig. 1D). On the contrary, keratinocytes from unaffected abdominal area (ANa) displayed similar levels of cadherin compared to control cells and no increase of vimentin.

Wnt proteins have a role in the regulation of cell adhesion, differentiation and EMT [32]. Ligands that inhibit phosphorylation of β-catenin by glycogen synthase kinase (GSK)-3β and its subsequent degradation are indicated as canonical (e.g., Wnt-1, 3a, 8, 10a), otherwise they are termed non-canonical (e.g., Wnt-4, 5a, 11). Activation of non-canonical Wnt signaling by ligands like Wnt-5a promotes several changes, such as protein kinase C activation and cytoskeletal rearrangements [32, 33].

ANp keratinocytes displayed higher expression of Wnt5a and lower levels of Wnt10a compared to Cp (Fig. 1E). Wnt3a was barely detectable in plantar keratinocytes of both control and patient, whereas Wnt4 did not vary. Interestingly, ANp keratinocytes expressed higher levels of the RSPO1 receptor Lgr5 compared to Cp cells.

Thus, keratinocytes from affected plantar hyperkeratotic skin of *RSPO1*-mutated patient display an EMT-like phenotype.

### Keratinocytes from affected plantar hyperkeratotic skin of *RSPO1*-mutated patient manifest invasiveness properties

**Fig. 2:**
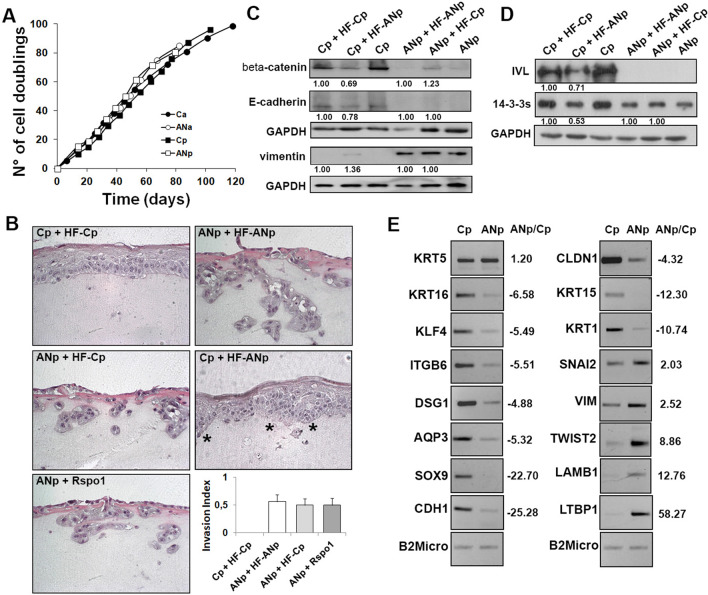
Keratinocytes from affected plantar hyperkeratotic skin of *RSPO1*-mutated patient display invasiveness properties. **A** Primary keratinocytes from affected plantar (ANp) and unaffected abdominal (ANa) skin of *RSPO1*-mutated XX-sex reversed patient AN, and from plantar (Cp) and abdominal (Ca) skin of aged-matched unrelated donor were serially cultivated. The cumulative number of cell generations per passage was plotted against the total time in culture. **B** Representative images of H&E of 3D organotypic skin models generated by seeding primary keratinocytes ANp or Cp on a matrix incorporating dermal fibroblasts from patient plantar skin (HF-ANp) or from control (HF-Cp) exposed at the air‒liquid interface to promote full epidermal differentiation and stratification. Models with ANp keratinocytes and fibroblasts were also treated with recombinant Rspo1 protein (ANp + RSPO). Relative Invasion Index values is reported (*n* = 10). **C**, **D** Immunoblots of β-catenin, E-cadherin, vimentin, 14–3-3 sigma and involucrin (IVL) in ANp or Cp keratinocytes co-cultured with irradiated PPK ANp-HFs, Cp-HFs and standard feeder-layer of 3T3-J2 cells. Densitometric values were normalized to GAPDH levels and expressed as fold change. **E** RT-PCR assay on ANp and Cp samples of selected differentially expressed genes. Densitometric values were normalized to Beta2 microglobulin levels and expressed as ratio ANp/Cp

EMT is associated with the conversion of early stage tumours into invasive malignancies [31]. To investigate whether *RSPO1* mutations could increase keratinocyte proliferative potential or induce immortalization, primary keratinocytes were serially cultivated. However, cultures from patient and control displayed a similar in vitro lifespan and underwent replicative senescence (Fig. 2A). Specifically, ANp and ANa keratinocytes underwent 75.07 and 75.04 cell doublings, respectively. Cp and Ca cells underwent 89.2 and 98.81 cell doublings, respectively.

The invasiveness of PPK keratinocytes was investigated in a three-dimensional organotypic skin model [34]. Cp were able to properly differentiate and reconstitute a stratified squamous epithelium that also includes a horny layer when seeded on a matrix containing fibroblasts (HF-Cp) from the same healthy donor (Fig. 2B, Cp + HF-Cp). Keratinocytes of the basal layer showed the normal columnar shape. On the contrary, ANp keratinocytes cultured in the presence of patient fibroblasts (HF-ANp) formed a completely disorganized epithelium which was composed of two–three cell layers only, lacked the basal cell regular arrangement and columnar shape and contained eosinophilic parakeratotic cells instead of anucleated corneocytes of the horny layer (ANp + HF-ANp). Of note, ANp keratinocytes were able to invade the matrix and this behaviour was not inhibited by the presence of normal Cp fibroblasts in the matrix (ANp + HF-Cp).

To study in-depth the influence of *RSPO1*-mutated fibroblasts on keratinocyte behaviour, keratinocytes were cultivated on feeder-layer composed by irradiated HF-ANp or HF-Cp fibroblasts. Standard feeder layer of 3T3-J2 was used as control. EMT-like morphology of ANp keratinocytes was maintained in the presence of both fibroblast types (not shown). Indeed, the expression of E-cadherin and vimentin did not vary (Fig. 2C, compare ANp + HF-ANp vs ANp + HF-Cp and ANp) although a slight β-catenin increase was observed in ANp + HF-Cp condition. In addition, HF-Cp fibroblasts were not able to rescue differentiation impairment as indicated by involucrin and 14–3-3 sigma expression (Fig. 2D, compare ANp + HF-ANp vs ANp + HF-Cp and ANp). Interestingly, Cp keratinocytes co-cultured with ANp fibroblasts displayed a moderate reduction of β-catenin, E-cadherin, involucrin and 14–3-3 sigma expression in concomitance with a slight increase of vimentin (Fig. 2C, D, compare Cp + HF-ANp vs Cp + HF-Cp and Cp). Although Cp keratinocytes did not show an EMT-like phenotype when co-cultured with HF-ANp fibroblasts (not shown), they reconstituted a disorganized epithelium marked by the lack of basal keratinocyte regular columnar shape and by the presence of nuclear remnants in cells of the uppermost layers (Fig. 2B, Cp + HF-ANp). These features are indicative of a hyperproliferative and dedifferentiated tissue. Of note, this altered epidermis focally invaded the matrix (Fig. 2B, Cp + HF-ANp, asterisks).

Thus, keratinocytes from affected plantar hyperkeratotic skin of *RSPO1*-mutated patient are endowed with invasiveness properties. Normal human control plantar fibroblasts are not able to revert the EMT-like phenotype of PPK keratinocytes. Importantly, *RSPO*-mutated PPK fibroblasts are capable to promote the dedifferentiation process and invasiveness of control plantar keratinocytes.

### The expression of genes involved in cell–cell adhesion, keratinocyte differentiation and extracellular matrix organization is perturbed in keratinocytes from affected plantar hyperkeratotic skin of *RSPO1*-mutated patient

The epidermis is a stratified squamous epithelium composed of a proliferative compartment (basal layer) and a multi-stage differentiation compartment (spinous, granular and horny layers) [35, 36]. Once keratinocytes detach from the basement membrane and move upwards, they change their gene expression profile under the control of p63 and other transcription factors and enter the differentiation process. Cornification is a unique form of terminal differentiation and programmed cell death leading to the formation of the outermost skin barrier as well as of hair and nails. The cell–cell contact is also important for the maintenance of the differentiated phenotype [37, 38]. The different epidermal layers can be distinguished by specific morphological features and expression markers [35, 36]. Palmoplantar epidermis is characterized by a very thick horny layer, the presence of a translucent layer (*stratum lucidum*) between the horny and granular layer, prominent rete ridges, the expression of keratin 9 (K9), a lower density of melanocytes and the absence of hair follicles. The stratum lucidum is a thin clear layer composed by dead keratinocytes densely packed with a lipoprotein that provides a barrier to water [39].

**Fig. 3:**
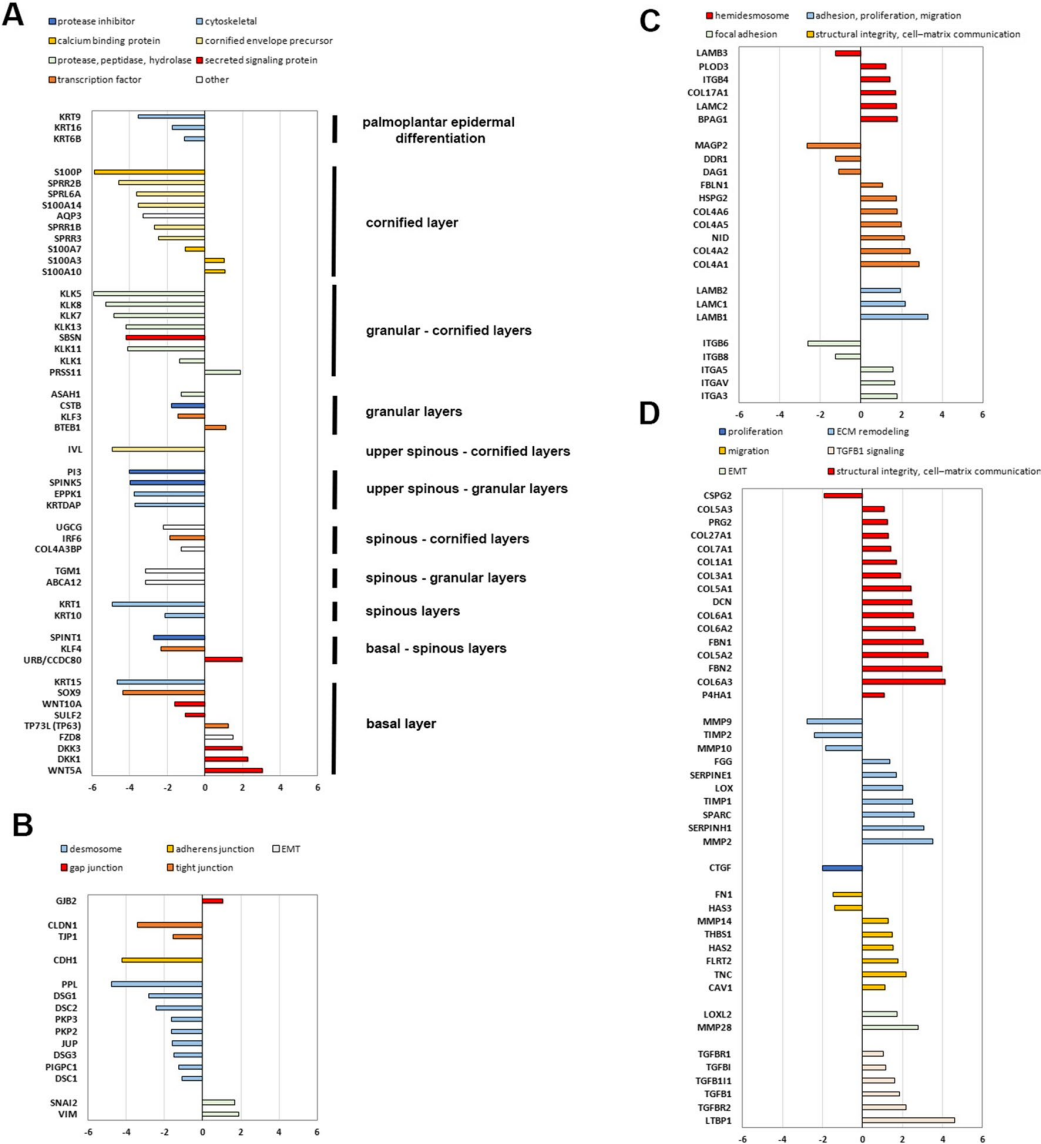
Differentially expressed genes in affected plantar hyperkeratotic skin of *RSPO1*-mutated patient vs plantar control. The expression level changes (Log2) of GO-enriched genes identified in primary keratinocytes from affected plantar (ANp) skin of *RSPO1*-mutated XX-sex reversed patient AN vs plantar (Cp) skin was shown. **A** Genes related to keratinocyte differentiation: the gene identity is listed on the left and localization by epidermal layers on the right. Protein function is reported in the upper legend. **B** Genes related to cell–cell junction: the gene identity is listed on the left and protein function in the upper legend. **C** Genes related to cell–matrix adhesion: the gene identity is listed on the left and protein function in the upper legend. **D** Genes related to ECM: the gene identity is listed on the left and protein function in the upper legend. Gene names corresponding to gene symbols are indicated in Additional file 1: Table S4

To unravel more deeply the cell–cell adhesion and differentiation processes, the transcriptome of ANp and Cp keratinocytes was compared by microarray hybridization. Differentially expressed genes are listed in Additional file 1: Table S1 and S2. Additional file 1: Table S3 summarizes the 1747 differentially expressed genes with detection *p* value < 0.05 in both samples and a > 2.0 or < 0.5 ANp/Cp fold change. Genes in Additional file 1: Table S3 were filtered for Gene Ontology (GO)-annotated genes and clustered according to biological processes. The most significantly modulated biological processes are reported in Table 1. Individual identification of GO-enriched genes is shown in Fig. 3. A synthetic description of the function of each protein and main expression pattern is indicated in Additional file 1: Table S4.

**Table 1:** Differentially expressed genes in affected plantar hyperkeratosic skin of RSPO1-mutated patient vs plantar control clustered into biological processes

GO biological process	Nr. genes	Fold Enrichment	*P* value	FDR	Up-regulated genes (fold change > 2.0)	Down-regulated genes (fold change < 0.5)
Intermediate filament bundle assembly	4/7	10.51	1.74E-03	2.98E-02	NEF3, NEFL	PKP2, EPPK1
Hemidesmosome assembly	7/12	9.19	2.02E-04	5.16E-03	ITGB4,LAMC1,LAMC2,BPAG1 (DST), COL17A1,CD151	LAMB3
Collagen-activated tyrosine kinase receptor signaling pathway	6/12	9.19	2.02E-04	5.16E-03	COL1A1,COL4A5,COL4A2,COL4A1, COL4A6	DDR1
Basement membrane assembly	5/10	9.19	7.04E-04	1.44E-02	LAMB1, PLOD3, LAMB2, PRG2 (PXDN)	LAMB3
Regulation of transforming growth factor beta activation	4/8	9.19	2.50E-03	4.01E-02	LTBP1, ITGAV	ITGB6, ITGB8
Desmosome organization	6/10	9.19	7.04E-04	1.44E-02	SNAI2	PKP2, PKP3, JUP, LBP-32 (GRHL1), PIGPC1(PERP)
Regulation of keratinocyte migration	6/14	7.88	3.86E-04	8.75E-03	HAS2, MAP4K4	MAPRE2, EPB41L4B, MMP9, EPPK1
Positive regulation of keratinocyte migration	5/12	7.66	1.33E-03	2.40E-02	HAS2, MAP4K4	MAPRE2, EPB41L4B, MMP9
Collagen-activated signaling pathway	6/15	7.35	5.16E-04	1.12E-02	COL1A1,COL4A5,COL4A2, COL4A1, COL4A6	DDR1
Basement membrane organization	11/28	7.22	2.89E-06	1.29E-04	PLOD3,LAMC2,NID1,FLRT2,COL4A1, LAMB1, EXT1, CAV1, LAMB2, PRG2 (PXDN)	DAG1
Regulation of extracellular matrix disassembly	6/16	6.90	6.79E-04	1.41E-02	IL6, TGFB1, FSCN1,LRP1, ETS1	DDR1
Regulation of extracellular matrix assembly	5/14	6.57	2.29E-03	3.74E-02	TGFB1, TEM8, HAS2,	DAG1, SOX9
Negative regulation of keratinocyte proliferation	5/14	6.57	2.29E-03	3.73E-02	KLF9 (BTEB1), EFNB2, CD109, SNAI2	EPPK1
Positive regulation of keratinocyte differentiation	6/18	6.13	1.12E-03	2.09E-02	CYP27B1, ALOX15B, NCOA3,	PRKCH, TRIM16, FOXC1
Extracellular matrix assembly	10/32	5.75	4.16E-05	1.35E-03	TGFB1, LOX, LAMB1, PLOD3, HAS2, LAMB2, PRG2 (PXDN), EFEMP2	HAS3, LAMB3
Heterotypic cell–cell adhesion	11/36	5.62	2.05E-05	7.29E-04	ITGAV, ITGA5, PARVA, CD1D, CD58, JAM3	PKP2, DSC2, JUP, PIGPC1 (PERP), CXADR
Collagen fibril organization	15/50	5.52	8.09E-07	4.01E-05	P4HA1, SERPINH1, LOX, COL5A3, COL1A1, CRTAP, EXT1, PLOD3, TGFBR1, PRG2 (PXDN), LOXL2, COL3A1, COL5A2, COL5A1	FOXC1
Regulation of bicellular tight junction assembly	7/24	5.36	8.42E-04	1.66E-02	IKBKB, SNAI2, RUNX1	EPHA2, PRKCH, CLDN1, TJP1 (ZO-1)
Substrate adhesion-dependent cell spreading	16/56	5.25	6.05E-07	3.05E-05	PIK3R1, NRP1, PLEKHC1, BVES, LAMB1, LAMC1, LPXN, TEM8, PARVA, AXL, PXN, LAMB2, FER, ITGAV	LAMB3, FN1,
Cell adhesion mediated by integrin	7/25	5.15	1.03E-03	1.96E-02	ITGA5, ITGB4, ADAM17, FBN1, ITGAV	ITGB6, ITGB8
Establishment of skin barrier	7/25	5.15	1.03E-03	1.96E-02	MET	KRT16, CLDN1, UGCG, ABCA12, FAAH, KRT1
Positive regulation of transforming growth factor beta receptor signaling pathway	8/29	5.07	4.95E-04	1.08E-02	TGFB1I1, THBS1, ADAM17	CDKN2B, CITED2, RGS19IP1, GOT1, CDKN1C
Positive regulation of extracellular matrix organization	6/22	5.01	2.63E-03	4.18E-02	IL6, TGFB1, FSCN1,	SOX9, CFLAR, DAG1
Positive regulation of epithelial to mesenchymal transition	13/48	4.98	1.11E-05	4.35E-04	IL6, TGFB1,COL1A1,TGFBR2,TGFBR1, PLEKHC1, NMA, LOXL2,TGFB1I1	SERPINB3, TIAM1, TCF7L2, FOXC1
Regulation of extracellular matrix organization	11/42	4.82	6.77E-05	2.03E-03	IL6, TGFB1,LRP1,HAS2, FSCN1, ETS1, TEM8	SOX9, DAG1, DDR1, CFLAR
Positive regulation of epidermis development	8/31	4.75	7.19E-04	1.47E-02	CYP27B1, ALOX15B, NCOA3,	PPARD, PRKCH, KRT10, TRIM16, FOXC1
Regulation of keratinocyte differentiation	11/42	4.38	2.80E-04	6.73E-03	CYP27B1,TP63,CD109, RUNX1, NCOA3,ALOX15B	AQP3, PRKCH, SERPINB13, FOXC1, TRIM16
Regulation of transforming growth factor beta production	9/38	4.35	5.79E-04	1.23E-02	ATF2, LTBP1, THBS1, FBLN1, ITGAV	ITGB6, ITGB8, FN1, CD24
Positive regulation of epithelial cell migration	36/152	4.35	7.35E-12	8.64E-10	ITGA3, TGFB1, SPARC, THBS1, AKT3, WNT5A, NRP1, RRAS,ARHB, HAS2, ADAM17, CALR, TGFBR2, MAP4K4, HBP17, ETS1, VEGFC, PKD2, MET, GRN	CTSH, SOX9, HSPB1, RAB11A, ANXA3, PIK3C2A, RAB25, MMP9,EPB41L4B, ANXA1, EDN1, FGF1, MAPRE2, PIK3CB, SNK, EGF
Extracellular matrix organization	85/363	4.31	5.46E-26	3.77E-23	P4HA1, TNC, ITGA5, TGFB1, MMP28, ITGB4, SERPINH1, COL27A1, COL4A6, SERPINE1, LOX, COL5A3, LAMB1, COL6A3, LAMC1, PTX3, COL4A5, COL1A1, TIMP1, CRTAP, APP, NID1, URB, EXT1, COL6A2, FBN2, HSPG2, PLOD3, THBS1, TGFBR1, HAS2, COL4A1, ITGA3, DCN, CAV1, LAMB2, CD44, COL7A1, LAMC2, BMP1, COL6A1, PRSS11, PRG2 (PXDN), LOXL2, FLRT2, ST7, EFEMP2, COL3A1, COL4A2, TGFBI, JAM3, SPARC, CAPNS2, COL5A2, FGG, FBN1, FBLN1, ITGAV, COL17A1, MMP14, COL5A1, MMP2	CDH1, ITGB6, ITGB8, DAG1, KLK7, SOX9, MMP9, LAMB3, FN1, SPINK5, DDR1, KLK5, FOXC1, HAS3, CSPG2, MMP10, DNAJB6, TIMP2, CTGF, SULF2, MAGP2, SPINT1, BIGM103
Cell-substrate junction assembly	10/43	4.28	3.29E-04	7.65E-03	ITGA5, PLEKHC1, CD151, ITGB4, LAMC1, LAMC2, COL17A1	LAMB3, FN1, ARHD
Regulation of epidermis development	15/69	4.00	2.30E-05	8.07E-04	DLL1, CYP27B1, ALOX15B, NCOA3, CD109, RUNX1	PRKCH, KRT10, SERPINB13, AQP3, TRIM16, FOXC1, HES1, MAFF, PPARD
Cornification	24/113	3.91	1.33E-07	7.62E-06		JUP, KLK13, KRT6B, SPRR2B, PKP2, TGM1, PKP3, KRT16, SPRR1B, KRT10, DSC1, IVL, PPL, KRT15, PI3, PIGPC1, DSC2, SPRR3, K6HF, KRT1, DSG1, SPINK5, DSG3, KLK5
Negative regulation of cell–matrix adhesion	8/38	3.87	2.22E-03	3.65E-02	PIK3R1, SERPINE1, THBS1, LRP1, CDKN2A, MAP4K4, MMP14	RASA1
Regulation of epidermal cell differentiation	13/62	3.86	1.10E-04	3.09E-03	DLL1, CYP27B1, ALOX15B, NCOA3, CD109, RUNX1	PRKCH, HES1, MAFF, SERPINB13, AQP3, TRIM16, FOXC1
Negative regulation of cell-substrate adhesion	13/63	3.79	1.26E-04	3.49E-03	PIK3R1, SERPINE1, COL1A1, THBS1, LRP1, CDKN2A, MAP4K4, FBLN1, MMP14, LGALS1	TACSTD2, RASA1, GBP1
Extracellular matrix disassembly	13/66	3.62	1.89E-04	4.89E-03	LAMC1, TIMP1, CD44, BMP1, PRSS11, CAPNS2, MMP14, MMP2	KLK7, MMP9, TIMP2, MMP10, KLK5
Keratinocyte differentiation	34/269	2.32	2.00E-05	7.13E-04	TXNIP, WNT5A, PSAP	EPHA2, JUP, KLK13, IRF6, KRT6B, SPRR2B, PKP2, TGM1, PI3, PKP3, KRT16, ASAH1, SPRR1B, KRT10, S100A7, DSC1, IVL, UGCG, PPL, KRT15, PIGPC1, DSC2, ABCA12, SPRR3, K6HF, KRT1, DSG1,SPINK5, DSG3, KLK5, ANXA1
Cell junction organization	61/491	2.28	2.35E-08	1.55E-06	TNC, GJB2, ITGA5, TGFB1, PRNP, NFIA, NRP1, PLEKHC1, CD151, ITGB4, RAB7L1, DNER, LAMC1, COL4A5, APP, DBN1, EXT1, VMP1, WNT5A, SYNPO, FSCN1, COL4A1, ITGA3, LAMB2, PCDHGC3, MAP1B, LAMC2, FLNA, JAM3,, NEDD, SNAI2, WRB, NEFL, COL17A1, PAK2	CXADR, JUP, CDH1, PKP2, POF1B, IL1RAP, PKP3, SPTBN2, INADL, EFNB2, CLDN1, TRAD, ARHD, KLK8, PTPRF, LNIR, PIGPC1, CAST, F2RL1, UNC13, LAMB3, DKK1, DSG1, TJP1, PRKCI

In agreement with morphological and immunophenotypic changes, dramatic differences between Cp and ANp keratinocytes were detected for terminal differentiation markers and proteins involved in cornification process and establishment of skin barrier. Expression of selected transcripts was verified by RT-PCR (Fig. 2E). Keratins of suprabasal layers (i.e. K1 and K10), some keratinocyte differentiation regulators (IRF6, KFL3, KLF4, KRTDAP), most of proteins involved in the cornification process (e.g. calcium binding proteins, cornified envelop precursors, small proline-rich proteins, kallikreins, the enzyme transglutaminase 1, transporter proteins, proteases, peptidases, hydrolases and protease inhibitors) were highly down-regulated in ANp keratinocytes (Fig. 3A). Among keratins expressed in palmoplantar skin (K6a, K6b, K9, K16, K17), K9 is the only one entirely specific to the palm and sole [39]. Affected keratinocytes displayed a strong down-regulation of K9, K6b and K16 (Fig. 3A and Additional file 1: Table S4). Likewise, K17 was slightly reduced in ANp vs Cp cells (Additional file 1: Table S1). On the contrary, keratins of the basal layer K5 and K14 display similar expression levels in patient and control (Additional file 1: Table S1). K15, which is mainly involved in epithelial cell morphogenesis, was highly down-modulated in ANp keratinocytes. p63 (indicated as TP73L) was more expressed in ANp cells compared to Cp in keeping with western blot data. Among proteins of Wnt pathway, Wnt-5a, DKK1 and DKK3 were highly expressed in ANp keratinocytes whereas Wnt-10a and Sulf2, which increases Wnt3a expression [40], were less expressed.

ANp cells displayed a strong down-modulation of most key components of specialized cell–cell junctions, mainly desmosomes, supporting the morphological and immunophenotypic data (Fig. 3B and Additional file 1: Table S4). On the contrary, connexin 26, which is a gap junction component, was up-regulated. The repression of the epithelial phenotype with loss of cell–cell contact and activation of the mesenchymal phenotype during EMT involves master regulators, including SNAI1 (Snail), SNAI2 (Slug), TWIST and ZEB transcription factors. Transcriptional activity of Slug is also regulated by vimentin-ERK axis [31, 41]. Notably, the transcripts of vimentin and Slug were up-regulated in ANp keratinocytes (Fig. 3B and Additional file 1: Table S4). The EMT transcription factor Twist1 displayed similar expression levels in ANp and Cp cells (Additional file 1: Table S1). However, PCR data indicated an increased expression of Twist2 in ANp keratinocytes (Fig. 2E).

Some hemidesmosome and basal membrane components, involved in tissue structural integrity and cell–matrix communication, were up-regulated (Fig. 3C and Additional file 1: Table S4) in ANp keratinocytes. Specific basal membrane laminins modulate cellular phenotype and differentiation. The expression of laminin chains beta1 (*LAMB1*) and gamma1 (*LAMC1*) was highly increased in ANp keratinocytes. Notably, these chains are assembled in laminin-511 and overexpressed in several malignancies contributing to tumor dissemination and metastasis [42]. EMT process is associated to changes in cytoskeleton organization as well as focal adhesion turnover instrumental to cell motility and invasiveness [41]. In fact, the cells down-regulate some epithelial integrins, but activate the expression of others with key roles in EMT progression [31]. Accordingly, some integrins (alpha 3, alpha 5, alpha V), which constitute focal adhesions and bind specific ECM proteins, were increased in ANp keratinocytes (Fig. 3C and Additional file 1: Table S4). Few integrins, DDR1 and microfibril-associated glycoprotein-2 were down-modulated.

The ECM is composed of a variety of macromolecules that have an active and complex role in regulating the cell behavior. Remodeling of the ECM and changes of cell–matrix interactions are essential in the initiation and progression of EMT [43, 44]. Most proteins involved in ECM organization, mainly structural dermal integrity and cell–matrix communication, were highly expressed in ANp keratinocytes (Fig. 3D and Additional file 1: Table S4). Moreover, ANp keratinocytes displayed up-regulation of proteins involved in migration and ECM remodeling, indicating a strong and active reorganization of the extracellular microenvironment that can favor cellular invasiveness. Some up-regulated genes are also involved in EMT and TGF-beta signaling (Fig. 3D and Additional file 1: Table S4).

Thus, differences in the transcriptome of keratinocytes from affected plantar hyperkeratotic skin of *RSPO1*-mutated patient and plantar control were in line with our morphological, immunophenotypic and functional findings.

### *RSPO1*-mutated palmoplantar skin of the patient shows defects in differentiation and cell–cell adhesion

Besides PPK, AN patient showed a huge predisposition to cutaneous SCCs. Up to now, he developed 48 SCCs on his palms and soles. Specifically, 40 SCCs appeared on palms, including 24 on fingers, and 8 on soles. Among SCCs, 30 were in situ lesions and 18 were highly infiltrating tumors.

**Fig. 4:**
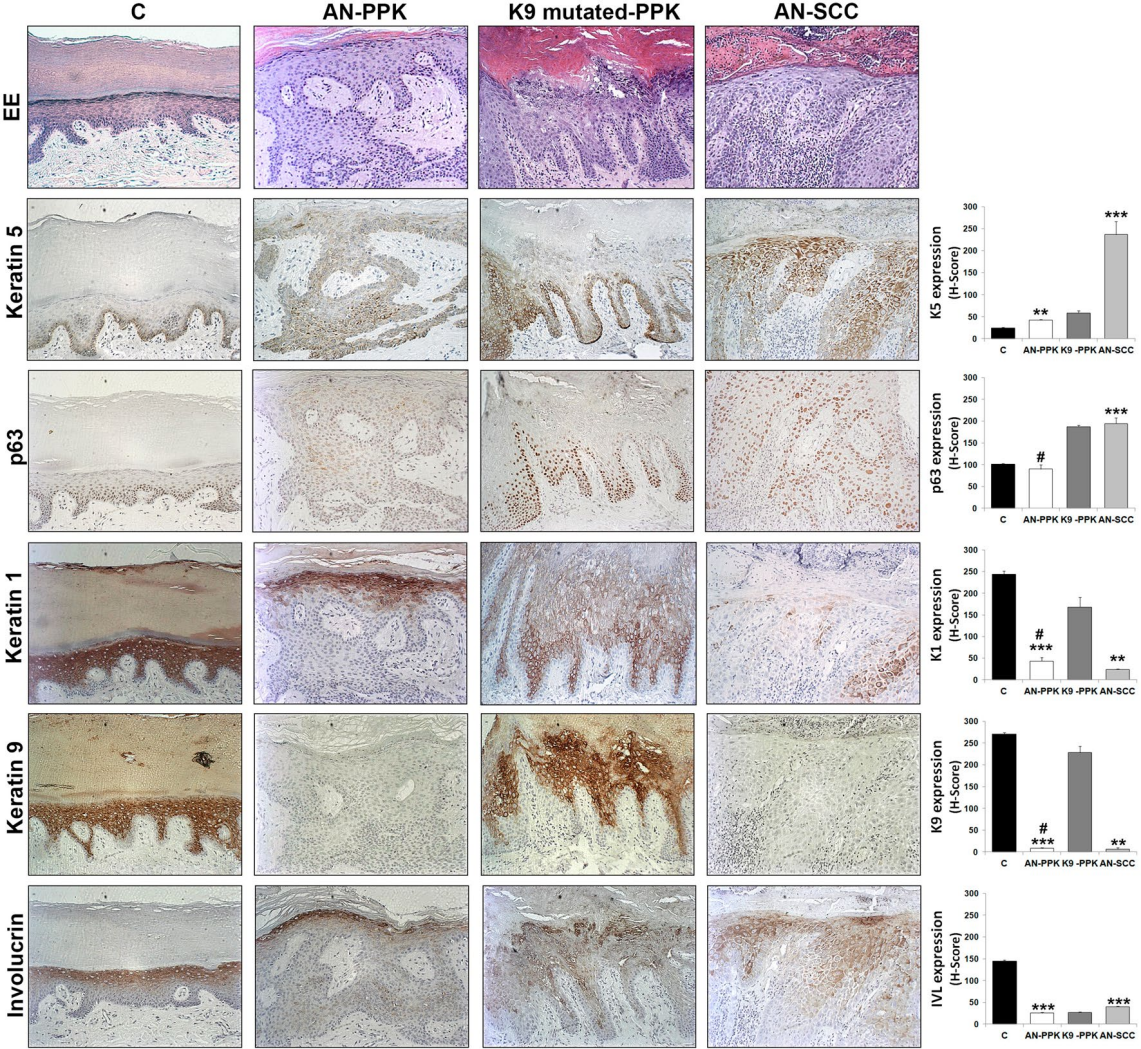
Impairment of keratinocyte differentiation in patient skin specimens. Paraffin-embedded specimens obtained from plantar skin (C), PPK (AN-PPK) and SCC (AN-SCC) areas of *RSPO1*-mutated XX-sex reversed patient, and PPK (K9-mutated -PPK) areas of patients suffering from epidermolytic PPK due to p.R162W mutation in *KRT9*, were stained with H&E. Specimens were immunostained with antibodies against keratin 5, p63, keratin 1, keratin 9 and involucrin. H-score of each specimen was calculated. Data are shown as mean ± SD. **P < 0.01, *** *P* < 0.001 compared to control, #*P* < 0.05 compared to K9-mutated-PPK

To further confirm gene expression data, we immunohistochemically compared paraffin-embedded plantar PPK skin and infiltrating SCC areas of the AN patient with plantar control skin and PPK from two patients with epidermolytic hyperkeratosis due to mutations in *KRT9* gene (Fig. 4). Among hereditary PPKs, non-syndromic isolated forms are characterized by a unique or predominant palmoplantar involvement since mutations affect structural proteins only expressed in the epidermis of palms and soles, such as K9. On the contrary, syndromic PPKs present additional extracutaneous manifestations, such as *RSPO1* mutated patients, and hyperproliferation of palms and soles may occur in response to the excessive mechanical stress to which these regions are exposed [16, 17].

Control plantar skin displayed a thicker cornified layer, more pronounced interdigitation between the epidermis and dermis, and the stratum lucidum just above the granular layer (Fig. 4, C, EE). PPK sections from AN patient revealed prominent orthokeratotic hyperkeratosis, hypergranulosis, irregular hyperplasia of the epidermis, focal dyskeratosis, elongation of rete ridges in the dermis and dermal scarring sclerosis (Fig. 4, AN-PPK, EE). PPK sections from patients with epidermolytic hyperkeratosis with mutant K9 showed hyperkeratosis and vacuolar degeneration of keratinocytes in the upper spinous and granular layers, where numerous large irregular granules were visible (Fig. 4, K9-mutated-PPK, EE). SCC sections from AN patient were characterized by hyperplasia of the epidermis, atypical and dyskeratotic keratinocytes invading the dermis and lymphocyte infiltration (Fig. 4, AN-SCC, EE).

**Fig. 5:**
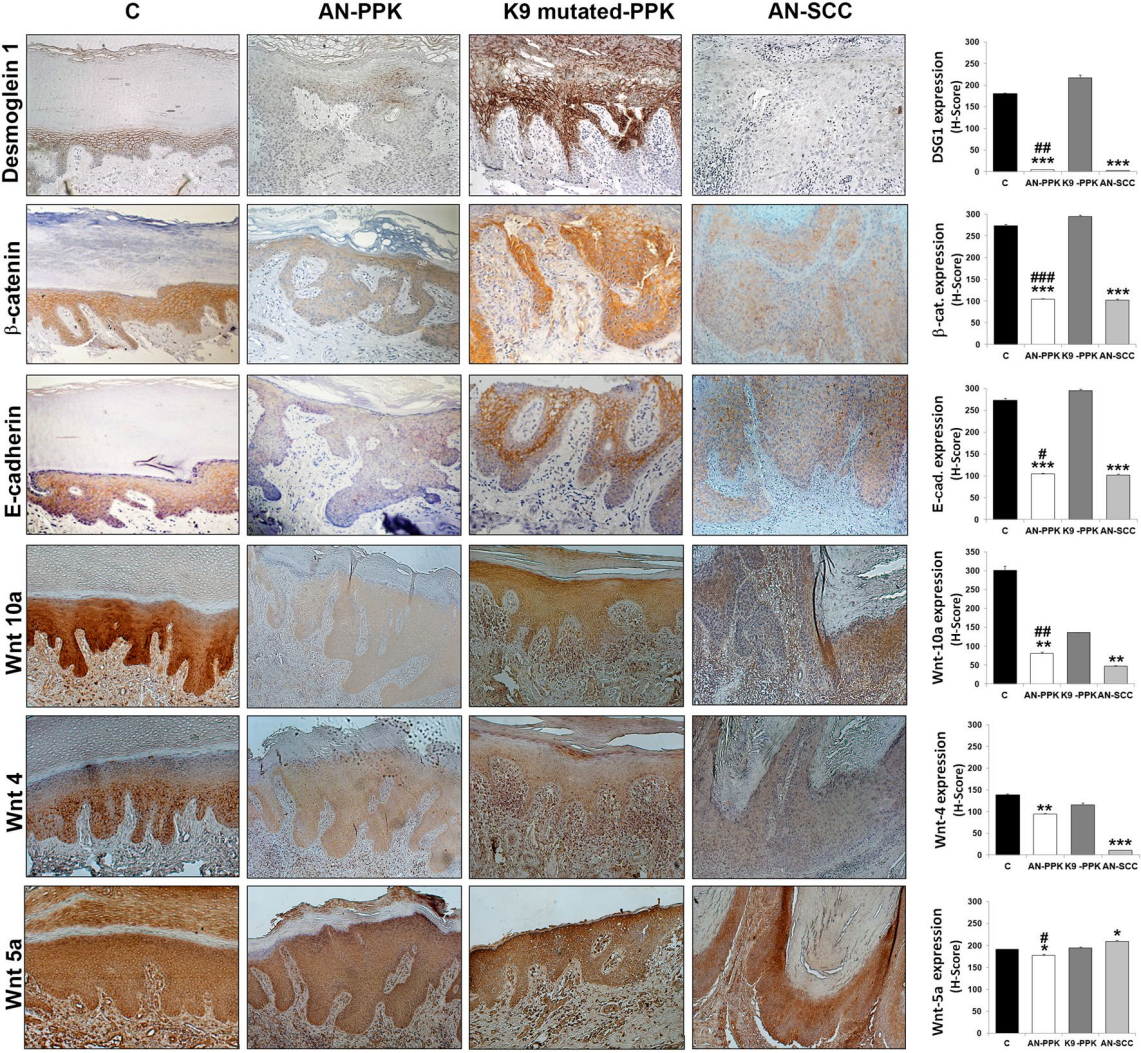
Impairment of cell–cell adhesion in patient skin specimens. Paraffin-embedded specimens obtained from plantar skin (C), PPK (AN-PPK) and SCC (AN-SCC) areas of *RSPO1*-mutated XX-sex reversed patient, and PPK (K9 mutated -PPK) areas of patients suffering from epidermolytic PPK due to R162W mutation in *KRT9*, were immunostained with antibodies against desmoglein 1, β-catenin, E-cadherin, Wnt-10a, Wnt-4 and Wnt-5a. H-score of each specimen was calculated. Data are shown as mean ± SD. *P < 0.05, *** *P* < 0.001 compared to control, #*P* < 0.05, ##*P* < 0.01, ###*P* < 0.001 compared to K9-mutated-PPK

As indicated by the H-score (Fig. 4), the expression of basal K5 was higher in PPK and SCC specimens from the patient compared to control. PPK areas of AN patient displayed p63 H-score similar to control samples. However, p63 was expressed only in keratinocytes of the basal and first suprabasal layers of control samples whereas it was scant though detectable in almost all epidermal cells of AN-PPK samples. p63 expression was significantly lower in AN-PPK compared to K9-mutated PPK. p63 was well-expressed in all keratinocytes of AN-SCC specimens and H-score was significantly higher compared to control. Keratin 1 (K1) was uniformly and strongly expressed in the cytoplasm of the suprabasal and upper layers of the control epidermis and was significantly reduced in AN-PPK and AN-SCC specimens. Its expression in PPK area of AN patient was lower than in patients with K9-mutation. Moreover, in AN-PPK specimens K1 was mainly present in upper layers, whereas in PPK with K9-mutation its expression was mainly in suprabasal layers. However, K1 was less expressed in the latter samples compared to control. Plantar epidermis expressed the specific suprabasal K9 similarly to K9-mutated PPK specimens. Notably, both AN-PPK and AN-SCC specimens did not express detectable levels of K9. Involucrin (IVL) was highly expressed in upper layers of the epidermis and significantly reduced in AN-PPK, K9-mutated PPK and AN-SCC specimens. Desmoglein 1 (Fig. 5) was regularly expressed along the membrane of keratinocytes of all suprabasal layers in plantar epidermis. It was strikingly reduced in AN-PPK and AN-SCC specimens and over-expressed in K9-mutated PPK samples. β-catenin and E-cadherin were regularly and intensely expressed at cell–cell contacts in control specimens. Their membrane expression was irregular and not uniform in K9-mutated-PPK. Both AN-PPK and AN-SCC specimens displayed a reduced and mainly cytoplasmic expression of β-catenin and E-cadherin. Notably, a nuclear staining of β-catenin was observed in keratinocytes of the tumour rim. Wnt10a was well expressed in plantar epidermis and significantly reduced in AN-PPK and AN-SCC specimens. Its expression in PPK area of AN patient was lower than in patients with K9 mutation. Wnt4 was more significantly expressed in plantar epidermis compared to AN-PPK and SCC samples. Wnt4 expression did not significantly vary between AN-PPK and K9-mutated-PPK. Wnt5a was well expressed in plantar epidermis. Its expression was slightly reduced in AN-PPK skin and significantly increased in AN-SCC specimens. Wnt5a expression did not significantly vary between plantar epidermis and K9-mutated-PPK.

Altogether these findings show that major alterations of keratinocyte differentiation and cell–cell adhesion are present in affected plantar hyperkeratotic skin of *RSPO1*-mutated patient. Of note PPK patient skin shares a similar pattern of biomarker expression with SCC specimens.

### Keratinocyte treatment with RSPO1 protein does not revert the EMT-like phenotype

**Fig. 6:**
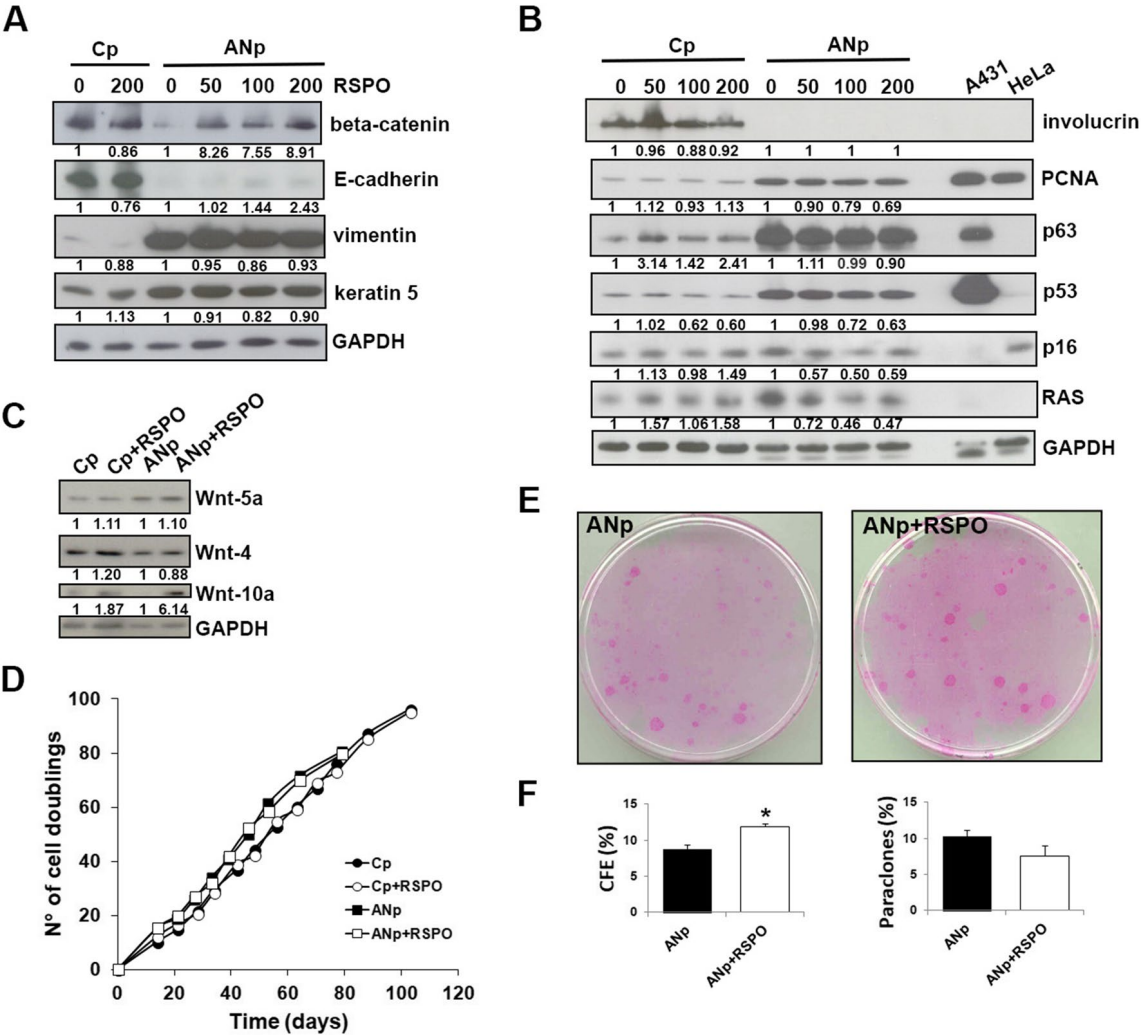
Keratinocyte treatment with Rspo1 protein does not revert the EMT-like phenotype**.** Primary keratinocytes from affected plantar (ANp) skin of *RSPO1*-mutated XX-sex reversed patient AN and from plantar (Cp) skin of aged-matched unrelated donor were treated with recombinant Rspo1 protein. **A**, **B** Immunoblots of β-catenin, E-cadherin, vimentin, keratin 5, involucrin, PCNA, p63, p53, p16, and RAS in ANp or Cp keratinocytes treated with several doses of Rspo1. Densitometric values were normalized to GAPDH levels and expressed as fold change. **C** Immunoblots of Wnt-5a, Wnt-4 and Wnt-10a in ANp or Cp keratinocytes treated with 200 ng/ml of Rspo1. Densitometric values were normalized to GAPDH levels and expressed as fold change. **D** ANp and Cp were serially cultivated in presence of 200 ng/ml of Rspo1 and compared to corresponding untreated culture. The cumulative number of cell generations per passage was plotted against the total time in culture. **E** Representative images of colony forming efficiency (CFE) of Rspo1-treated and untreated ANp cultures. D CFE values and percentage of aborted colonies (paraclones) in Rspo1-treated and untreated ANp cultures (*n* = 3). Data are shown as mean ± SD. *P < 0.05

In an attempt to reverse the EMT-like phenotype of ANp keratinocytes, they were treated with the recombinant RSPO1 protein. Although RSPO1 treatment induced β-catenin increase (Fig. 6A), the EMT-like morphology of these cells was neither reversed in culture (not shown) nor in the organotypic skin model (Fig. 2B, AN + RSPO1). Indeed, E-cadherin and vimentin expression did not change significantly after treatment with increasing doses of RSPO1 protein (Fig. 6A). Especially at highest dose, the RSPO1-treated ANp cells slightly reduced the expression of K5, PCNA and some proteins that play an important role in SCC promotion and invasion (p63, p53, p16 and RAS) (Fig. 6A, B). However, the differentiation marker involucrin, and Wnt4 and Wnt5a did not vary following treatment (Fig. 6B, C). Notably, Wnt10a expression was increased in ANp cells following RSPO1 treatment (Fig. 6C).

ANp cultures underwent a similar number of cell duplications at the end of their in vitro life span either in the presence of RSPO1 recombinant protein (82.52 cell doublings) or in untreated cultures (78.11 cell doublings) (Fig. 6D). Analogously, RSPO1 treatment did not affect the life span of control keratinocytes. Notably, the clonogenic ability of ANp cultured keratinocytes was clearly increased by RSPO1 treatment, as indicated by the higher CFE (Fig. 6E, F). The treatment induced only a slight decrease of paraclone percentage (Fig. 6F).

Thus, RSPO1 treatment does not restore epidermal differentiation or significantly modulate the EMT features of keratinocytes from affected plantar hyperkeratotic skin of *RSPO1*-mutated patient.

## Discussion

*RSPO1*-mutated XX-sex reversed AN patient suffers from PPK since birth, and also manifests sclerodactyly of the hands, nail dystrophies, and a strong predisposition to develop in adulthood malignant SCCs in PPK skin [3].

Here, we show that primary keratinocytes from PPK skin of this patient display impaired differentiation, EMT-like phenotype, and invasiveness properties. Importantly, PPK skin in patient’s specimens shows analogous features and these signs are more evident in SCC specimens.

### RSPO1 mutations and PPK

Palms and soles differ from other body sites in terms of clinical and histological appearance and response to mechanical stress. PPK is a common clinical sign of genetic and acquired diseases showing defects in many but often converging signalling pathways [16, 17]. Notably, impaired keratinocyte terminal differentiation is an early driver of PPK lesion onset and progression [45]. Accordingly, the epidermis of PPK area of AN patient is characterized by dedifferentiation features. Moreover, cultured keratinocytes isolated from PPK area were unable to stratify and undergo terminal differentiation in the organotypic skin model. In fact, they display strong down-regulation of most of genes encoding structural proteins mainly involved in epidermal cornification or coding for components of cell–cell junctions, specifically of the desmosomes. Interestingly, some of these genes, i.e. *KRT1, KRT9, KRT16, DSG1, DSC2* and *JUP*, are mutated in hereditary PPKs [16, 17], while the A*BCA12* gene is defective in harlequin ichthyosis characterized by the loss of the skin lipid barrier [46]. In AN patient, PPK might be due to developmental defects, consistent with the early appearance of the PPK phenotype soon after birth. Notably, K16 is a positive regulator of palmoplantar differentiation-associated K9 expression [45] and both proteins are highly down-regulated in ANp keratinocytes. Missense mutations at the *KRT16* locus can cause pachyonychia congenita-associated PPK or focal non-epidermolytic PPK. Moreover, complete loss of *KRT16* in mice impairs the differentiation of palmoplantar keratinocytes despite the up-regulation of many effectors of epidermal differentiation [45].

Furthermore, key differentiation-associated transcription factors (e.g. IRF6, KLF3, KLF4) are down-regulated in ANp keratinocytes. Of note, Notch1 and KLF4 are repressed by ΔNp63 whereas IRF6 enhances ΔNp63 protein degradation [47]. Accordingly, ANp keratinocytes display increased levels of p63.

Thus, *RSPO1* mutations perturb the balance between keratinocyte proliferation and differentiation.

### RSPO1 mutations and SCC susceptibility

Patients suffering from specific inherited forms of PPK (i.e. Huriez syndrome, Olmsted syndrome, a subset of patients suffering from keratitis-ichthyosis-deafness, hereditary dyschromatosis that is due to mutations in *SASH1* gene) are prone to SCC development [16, 17, 48]. Notably, cells from Olmest syndrome individuals mutated in *TRPV3* show an altered expression of genes involved in keratinocyte differentiation, cell–cell adhesion, proliferation and cell death in a pattern that is similar to what we observe in keratinocytes from the RSPO1-mutated patient [49]. However, PPK and risk for SCC are not always clinically linked. In fact, SCC predisposition is not observed in hereditary PPK due to K9 mutations, though K9-mutated keratinocytes show altered proliferation and differentiation. Differently from K9-mutated skin, the epidermis of AN-PPK presents anomalies in cell–cell contacts as those seen in SCC lesions. Likewise moderately differentiated SCC from general population, SCC from *RSPO1*-mutated patient display an increase of proliferation markers, a decrease of differentiation proteins, a reduction of cell–cell contact structural proteins and nuclear staining of β-catenin at the tumour rim.

The presence of SCCs at sites of scarring suggests that dysfunctional signaling between epithelial cells and their microenvironment may predispose to tumor development in adulthood. Notably, the EMT reprogramming is initiated and controlled by signaling pathways that respond to microenvironmental signals from neighboring cells or EMC [31].

Primary fibroblasts from PPK areas of *RSPO1*-mutated patients behave as cancer associated fibroblasts (CAFs), which promote collagen contraction and SCC matrix invasion. Moreover, fibroblasts display a sustained activation of TGF-β signaling with loss of its inhibitory feedback, and an increased secretion of matrix metalloproteases MMP1 and MMP3 [25]. Notably, ANp keratinocytes invade the matrix in the presence of AN fibroblasts. Although normal microenvironment has a tumor suppressive function [50], the co-culture of ANp keratinocytes with normal fibroblasts does not revert their EMT-like phenotype or reduce their invasion capability in the organotypic skin model. Moreover, even though treated ANp keratinocytes attempt to restore β-catenin expression, they do not recover normal E-cadherin and continue to express vimentin. Microarray data indicate that ANp keratinocytes themselves express high levels of several genes involved in EMT, ECM remodeling and invasion promotion. During EMT process, some components of cell–cell adhesion structures, such as β-catenin and plakoglobin, can switch from structural function to transcription factor activity, thereby contributing to changes in cell transcriptome. Phenotypic plasticity and ECM changes are also controlled by p63 [47] that is increased in ANp keratinocytes. Thus, keratinocytes obtained from PPK areas of the patient seem already committed to EMT process and able to activate ECM remodeling pathways that favor a microenvironment adapted for growth and spread of malignant cells irrespective of the presence of normal fibroblasts. Even recombinant RSPO1 addition does not recover the normal epithelial phenotype of *RSPO1*-mutated keratinocytes or counteract their invasion capability. Interestingly, *RSPO1*-mutated PPK fibroblasts attempt to promote proliferation and dedifferentiation of age-matched control plantar keratinocytes in the organotypic culture.

Therefore, dysregulation of signaling between keratinocytes and their microenvironment may promote developmental defects of skin and appendage. Chronic inflammation and microenvironment changes mediated by both skin cell types in PPK areas may have an additive role in causing SCC development and recurrence in *RSPO1*-mutated patient.

### RSPO1-mediated PPK, SCC and Wnt pathway

Wnt pathway is required for stem cell control and normal differentiation of the skin and its appendages, including nails [32, 33]. In fact, correct palmoplantar keratinization depends on intact canonical Wnt signaling that plays a key role in cell polarization instrumental for promoting and maintaining a differentiated phenotype [31, 51]. Deactivation of β-catenin led to differentiation impairment and thinner epithelium in the footpad and tongue in mice [52]. Moreover, decreased Wnt/β-catenin signaling due to *WNT-10a* loss-of-function mutations leads to a compensatory PPK as observed in odonto-onycho-dermal dysplasia and Schöpf-Schulz-Passarge syndrome. These patients have also hypo- to anodontia, nail abnormalities, and develop adnexal tumors, including basal cell carcinoma [16, 17]. As observed in *WNT-10a*-associated ectodermal dysplasias, the ANp keratinocytes may contribute to PPK development as a consequence of dysregulated canonical Wnt signaling and, in turn, loss of cell polarity. Notably, the nails of *RSPO1*-mutated patients are dystrophic as expected in the context of defective canonical Wnt signaling.

Deregulation of Wnt pathway has also a key role in tumorigenesis [32, 53]. Wnt pathway is tightly regulated through the dosage and timing of several ligands and receptors with partial overlapping functions. Wnt pathway activity is also finely-tuned by secretion of different activators and inhibitors: secreted RSPO1 reinforces canonical Wnt signaling whereas DKK1 inhibits canonical Wnt signaling by competitive binding to LRP5/6 [33].

Several changes might act synergistically to repress canonical and activate non-canonical Wnt signaling in ANp keratinocytes. β-catenin and Wnt10a are down-regulated and DKK1 is up-regulated in ANp keratinocytes compared to Cp suggesting that canonical Wnt/β-catenin pathway is inhibited. Conversely, non-canonical Wnt-5a levels are higher in ANp keratinocytes than Cp. Wnt-5a is up-regulated in SCC in general population. Notably, Wnt-5a hyperactivation is coupled to repression of canonical Wnt signalling and, in turn, contributes to dedifferentiation, EMT and tissue invasion. Concomitant Wnt-5a up-regulation and Wnt-4 down-regulation have been found in EMT of SCC [32, 54–57]. In keeping with these data, AN-SCC display significantly higher Wnt-5a and lower Wnt-4 levels compared to control skin. Unlike SCC from general population, AN-SCC express low levels of Wnt-10a.

### SCC therapeutic perspectives

Secreted RSPO proteins do not induce canonical Wnt signaling but markedly amplify the basal activity of Wnt ligands and act as self-renewal factors for Lgr5 + intestinal cells [58]. Accordingly, RSPO1 treated-ANp keratinocytes display an increase of clonogenic ability and reduced levels of proteins involved in cell-cycle regulation and cell senescence. However, RSPO1 treatment does not normalize epidermal differentiation or modulate the EMT features. Notably, Wnt-5a expression does not vary following treatment suggesting that the maintenance of its high levels could still induce EMT. Thus, Wnt-5a could be an interesting therapeutic target for counteracting the EMT-like phenotype. For instance, a Wnt-5a-derived hexapeptide, termed Box5, has been shown to antagonize Wnt-5a /Ca2 + signaling in head and neck SCCs [59]. Furthermore, Lgr5 itself could have a role in EMT-like phenotype retention since may exert opposing functions during the progression of different cancer types. RSPO1/Lgr5 axis activates Wnt/β-catenin signaling and promotes EMT and tumor formation in breast cancer and glioblastoma [60] whilst, by enhancing TGFβ-mediated growth inhibition and stress-induced apoptosis, it suppresses metastasis in colon cancer [24]. Furthermore, RSPO1 treatment of *RSPO1*-mutated fibroblasts induces anti-proliferative and anti-fibrotic effects but also aggravates their pro-inflammatory phenotype [25]. This finding may explain why RSPO1 treatment does not counteract the invasive capability of *RSPO1*-mutated keratinocytes [25].

Therefore, given the ubiquitous nature and pleiotropic effects of Wnt signaling, caution should be taken when designing therapeutic strategies. Solid investigations using preclinical models will help to clarify whether Wnt pathway proteins may represent suitable targets for future therapies in patients.

## Conclusions

Altogether our data indicate that patients with 46XX disorders of sexual development with *RSPO1* loss-of-function mutations are exposed to a high risk of SCC, mainly due to altered keratinocyte behaviour and pro-tumorigenic changes in the skin microenvironment of palmoplantar regions exposed to major mechanical force.

## Methods

### Patient samples

The *RSPO1* recessive mutation of patient AN (a 56-year-old male) was previously described [3] and found to be associated with complete female-to-male sex reversal, PPK and predisposition to SCC of palms and soles (Palmoplantar Hyperkeratosis—Squamous Cell Carcinoma of skin—Sex Reversal; OMIM #610,644).

Cultures of human primary keratinocytes and fibroblasts were established from hyperkeratotic non-tumoral plantar (ANp) and clinically normal abdominal (ANa) skin specimens of patient AN, and from abdominal (Ca) and plantar (Cp) skin of sex- and age-matched healthy donors (46 and 50-years old males). Skin biopsies were cultivated on a feeder layer of lethally irradiated 3T3-J2 murine fibroblasts as described by Rheinwald and Green [26].

Immunohistochemistry was carried out on formalin-fixed paraffin-embedded 3 μm thick sections of three AN-PPK, AN-SCC and palmo-plantar specimens of age-matched healthy donors. Specimens from two patients, a 43-year-old male and a 49-year-old female, suffering from epidermolytic PPK (Voerner type EPPK; OMIM #144,200) due to the p.R162W dominant mutation in the keratin 9 gene *KRT9* [61] were also analyzed.

### Cell cultures ad treatments

3T3-J2 cells (a gift from Prof Howard Green, Boston, MA, USA), primary human keratinocytes, and fibroblasts were grown as described previously [62]. SCC13 cells (a gift from James Rheinwald, Boston, MA, USA) were grown as described [28]. Ras-V12 (RAV-12) keratinocytes, which express the activated Ras oncogene, have been described in [29].

ANp or Cp keratinocytes were co-cultured with irradiated PPK fibroblasts (ANp-HFs), control plantar fibroblasts (Cp-HFs), or 3T3-J2 cells.

ANp and Cp keratinocyte cultures were also treated with increasing doses (50 ng/ml, 100 ng/ml and 200 ng/ml) of the recombinant RSPO1 protein (a gift from dr. Arie Abo, Nuvelo Inc, San Carlos, CA, USA).

### Colony forming efficiency (CFE) and life span cultures

Colony-forming efficiency (CFE) assay was performed as previously described [63]. CFE values were expressed as the ratio of the total number of colonies on the number of inoculated cells. The paraclone percentage was expressed as the ratio of aborted colonies to the total number of colonies.

Keratinocytes were serially cultured. The number of cell doublings was calculated according to the formula x = 3,322 log N/N0, where N is the total number of cells obtained at each step, and N0 is the total number of plated cells × colony-forming efficiency.

### Invasion assays

For three-dimensional organotypic culture generation, 5 × 10^5^ fibroblasts (ANp-HFs or Cp-HFs) were embedded in 1 ml matrix gel [64]. After 1 h at 37 °C, gels were overlaid with 5 × 10^5 ^primary keratinocytes (ANp or Cp). Three-dimensional models were lifted at the cell‒air interface 24 h later, cultured for 7 days, and then fixed. Quantification of the invasion index was assessed by measuring the specific area of paraffin-embedded sections of the three-dimensional models stained with H&E by ImageJ software. The invasion index was calculated as 1 − (noninvading area/total area) [64]. These co-culture models were also treated with the recombinant RSPO1 protein (200 ng/ml).

### Immunoblot analysis

Subconfluent keratinocyte extracts were prepared as previously described [63] and analyzed by 7.5–12.5% SDS polyacrylamide gel electrophoresis. Immunoblotting was performed as described previously [63].

The following primary antibodies were utilized: anti-PCNA (PC10), anti-p63 (4A4), anti-p53 (DO-1), anti-p16 (N20), anti-14–3-3 sigma (N14), anti-GAPDH (FL-335), anti-β-Catenin (E-5), anti-Wnt4, anti-Wnt-10a, all from Santa Cruz Biotechnology Inc., Santa Cruz, CA, USA; anti-involucrin (SY5) from Sigma-Aldrich, St. Louis, MO, USA; anti-vimentin (RV202) and anti-E-cadherin (36/E-Cadherin) from BD Biosciences, Franklin Lakes, NJ, USA; anti-cytokeratin 5 (EP1601Y) from Epitomics, Inc., Burlingame, CA, USA; anti-LGR5, anti-WNT-5a and anti-WNT-3a Origene Technologies, Herford, Germany).

Protein levels were evaluated by densitometric analysis by a GS-710 scanner and QuantityOne software (Bio-Rad) and then normalized to GAPDH protein levels.

### Microarray analysis

30 ug of total RNA from ANp and Cp keratinocytes were used for microarray analysis. Analysis has been performed at John Innes Center (UK) using the HG133 set (A and B) Chip from Affymetrix following the supplier instruction. Differentially expressed genes with detection *p* value < 0.05 in both samples and a > 2.0 or < 0.5 ANp/Cp fold change were filtered for Gene Ontology (GO)-annotated genes and clustered in biological processes.

Semi-quantitative PCR have been performed. For each gene tested PCR amplification was stopped at exponential level.

### Immunohistochemistry

Immunohistochemistry was carried out as described previously [63]. The anti-K9 (Ks 9.70/Ks 9.216) was from Progen Biotechnik GmbH, Heidelberg, Germany; anti-K5 (EP1601Y) was from Epitomics, Inc., Burlingame, CA, USA; anti-K1 (34βB4) was from Sanbio B.V. Uden, The Netherlands.

Immunohistochemistry results were evaluated by a semiquantitative approach used to assign an H-score to each specimen [65]. Briefly, staining intensity (0, 1 + , 2 + , or 3 +) was determined for each cell in a fixed field. The H-score was assigned using the following formula: [1 × (% cells 1 +) + 2 × (% cells 2 +) + 3 × (% cells 3 +)].

### Statistical analysis

Data were expressed as mean ± SD. Statistical analysis was performed using Student’s *t*-test. Differences were considered statistically significant at *P* < 0.05.

## Supplementary Information

**Additional file1.** **Table** **S1**. Differentially expressed genes in affected plantar hyperkeratosic skin of RSPO1-mutated patient vs plantar control - Affimetrix microarrays U133a Raw data).Values in the Signal column reflect intensity. The Detection column assigns a call of Present, Absent, or Marginal to each probe set and the “Detection p-value” column provides an assessment of statistical significance of each call. The “Descriptions” column provides summary information about each transcript. **Table** **S2**. Differentially expressed genes in affected plantar hyperkeratosic skin of RSPO1-mutated patient vs plantar control - Affimetrix microarrays U133b Raw data).Values in the Signal column reflect intensity. The Detection column assigns a call of “Present, Absent, or Marginal to each probe set and the Detection p-value column provides an assessment of statistical significance of each call. The Descriptions column provides summary information about each transcript. **Table** **S3**. Differentially expressed genes in affected plantar hyperkeratosic skin of RSPO1-mutated patient vs plantar control. **Table** **S3** summarizes 1747 differentially expressed genes with detection p value <0.05 in both samples and a >2.0 or <0.5 ANp/Cp fold change. **Table** **S4**. Individual identification of GO-enriched genes differentially expressed genes in affected plantar hyperkeratosic skin of RSPO1-mutated patient vs plantar control.Genes in Supplementary **Table** **S3** were filtered for Gene Ontology (GO)-annotated genes and clustered in biological processes. Individual identified GO-enriched genes are reported.

## Data Availability

The datasets supporting the conclusions of this article are available in the Mendeley repository, Dellambra, Elena (2022), “RSPO1-mutated keratinocytes from palmoplantar keratoderma display impaired differentiation, alteration of cell–cell adhesion, EMT-like phenotype and invasiveness properties:”, Mendeley Data, v1http://dx.doi.org/10.17632/28kxws739v.1."

## Abbreviations

RSPO: R-spondin
PPK: Palmoplantar keratoderma
SCC: Squamous cell carcinoma
EMT: Epithelial-mesenchymal transition
CAF: Cancer-associated fibroblasts
HF: Human fibroblasts
ANp: PPK area of the *RSPO1*-mutated patient
Cp: Plantar area of control
ANa: Abdomen area of the *RSPO1*-mutated patient
Ca: Abdomen area of control
